# Early inoculation with caecal fermentation broth alters small intestine morphology, gene expression of tight junction proteins in the ileum, and the caecal metabolomic profiling of broilers

**DOI:** 10.1186/s40104-019-0410-1

**Published:** 2020-01-15

**Authors:** Yujie Gong, Wenrui Xia, Xueting Wen, Wentao Lyu, Yingping Xiao, Hua Yang, Xiaoting Zou

**Affiliations:** 1 State Key Laboratory for Quality and Safety of Agro-products, Institute of Quality and Standards for Agro-products, Zhejiang Academy of Agricultural Sciences, Hangzhou, 310021 China; 2 Key Laboratory for Molecular Animal Nutrition of Ministry of Education, Feed Sciences Institute, College of Animal Sciences, Zhejiang University (Zijingang Campus), Hangzhou, 310058 China

**Keywords:** Broilers, Early inoculation, Gene expression, Intestine morphology, Metabolomics profiling

## Abstract

**Background:**

The establishment of stable microbiota in early life is beneficial to the individual. Changes in the intestinal environment during early life play a crucial role in modulating the gut microbiota. Therefore, early intervention to change the intestinal environment can be regarded as a new regulation strategy for the growth and health of poultry. However, the effects of intestinal environmental changes on host physiology and metabolism are rarely reported. This study was conducted to investigate the effects of early inoculation with caecal fermentation broth on small intestine morphology, gene expression of tight junction proteins in the ileum, and cecum microbial metabolism of broilers.

**Results:**

Our data showed that early inoculation with caecal fermentation broth could improve intestine morphology. The small intestine villus height was significantly increased (*P* < 0.05) in the intervened broilers compared to the control group, especially on day 28. A similar result was observed in the ratio of villus height to crypt depth (*P* < 0.05). Meanwhile, we found early inoculation significantly increased (*P* < 0.05) the expression levels of zonula occludens-1 (*ZO1*) on days 14 and 28, claudin-1 (*CLDN1*) on day 28, whereas the gene expression of claudin-2 (*CLDN2*) was significantly decreased (*P* < 0.05) on days 14 and 28. Gas chromatography time-of-flight/mass spectrometry (GC-TOF/MS) technology was further implemented to systematically evaluate the microbial metabolite profiles. Principal component analysis (PCA) and orthogonal partial least squares discriminant analysis (OPLS-DA) displayed a distinct trend towards separation between the fermentation broth group (F group) and the control group (C group). The differentially expressed metabolites were identified, and they were mainly functionally enriched in beta-alanine metabolism and biosynthesis of unsaturated fatty acids. In addition, 1,3-diaminopropane was selected as a key biomarker that responded to early inoculation with caecal fermentation broth.

**Conclusions:**

These results provide insight into intestinal metabolomics and confirm that early inoculation with caecal fermentation broth can be used as a potential strategy to improve intestinal health of broilers.

## Background

The gastrointestinal tract (GIT) of poultry harbors complex and highly diversified communities of microorganisms, which collectively fulfill crucial roles in host physiology and health [1, 2]. In addition to the local effect within the GIT, gut microbiota can also have a profound effect on the host through microbe-derived metabolites [3]. Gut microbiota provide metabolites to the host in the form of fermentation end-products and other secreted products such as lipids, carbohydrates, organic acid, amino acids and other nutrients [4], which could be supplied throughout the body to meet the host nutrient and energy needs [5]. Therefore, in a sense, the significance of microorganisms to host health can be explained by their metabolic potential.

The environment and diet during animal feeding are the main factors in modulating the gut microbiota. Accumulating evidence demonstrates that diet supplemented with probiotics [6], organic acids [7], and exogenous enzyme [8, 9] can increase the abundance of intestinal beneficial bacteria, which have a protective role as the first line of defense against pathogenic bacteria in addition to assistance in specific metabolism and gut structure integrity [10]. However, there is a lack of knowledge regarding the regulatory mechanism of the microenvironment on the intestinal microbiota of poultry. Although our previous study has shown that shaping a specific gut environment through early intervention can alter the colonization and development of caecum microbiota in broilers, resulting in a reduction in the relative abundance of enteric pathogen and an increase of beneficial bacteria [11], the downstream effects caused by altering the structure of the gut microbiome on host physiology still need to be further explored [3]. It has been reported that the regulation mechanism of intestinal microbiota on host overall health primarily depends on microbial metabolism [12, 13]. For example, many studies have demonstrated that host inflammatory diseases are closely related to microbial metabolic disorders [14, 15]. Microbiota metabolites, as one of the most predominant connections between gut microbiota and the host, have crucial biological significance. Certainly, in addition to metabolic function, gut microbiota are also indispensable contributors to the formation and development of gut structure and morphology [16]. Complete intestinal structure could guarantee the function of intestinal mucosal barrier, which is usually reflected by villus length and crypt depth [17]. On the other hand, tight junction proteins, as one of the most considerable components of the intestinal barrier, play a crucial role in intestinal function [18, 19]. Therefore, intestine morphology and gene expression of tight junction proteins are generally regarded as pivotal indicators of intestinal health.

Here, we present a study aimed at investigating the effect of environmental factors on the gut microbiota metabolism of broilers, by orally administering fermentation broth at the early stage of life. Gas chromatography time-of-flight/mass spectrometry (GC-TOF/MS)-based cecal metabonomic method was used to identify the metabolomics profiles. The differentially expressed metabolites and their involved metabolic pathways were characterized. In addition, small intestine morphology and expression levels of tight junction-proteins in the ileum were determined.

## Materials and methods

### Preparation of the caecal fermentation broth

In the present study, caecal fermentation broth was used as the inoculum for early inoculation, the preparation of which was concretely described in our previously published article [11]. A single-stage chemostat, as an *in vitro* bionic system, was used to produce the fermentation broth. First, caecal content from the selected donor chicken was thoroughly mixed with sterile phosphate buffered saline (PBS) to form a 10% suspension. Subsequently, the resulting suspension was injected into the chemostat and fermented continuously for 11 days. Finally, fermentation broth from the 11^th^ day was used to inoculate chicks.

### Animals and experimental design

A total of 120 one-day-old broiler chicks purchased from a local commercial hatchery were randomly divided into 2 groups with 4 replications and 15 birds per replicate, including 2 treatments: chicks in the experimental group were given 0.5 mL of fermentation broth orally within 2 h after hatching. In turn, chicks in the control group received the same amount of sterile PBS at the corresponding time. All experimental animals were raised in an environmentally controlled house in Zhejiang Academy of Agricultural Sciences, where the temperature of the first week was constant at 35 °C, and then lowered 3 °C weekly until the temperature reached 26 °C. The broilers received no antibiotics or other additives throughout the experimental period.

### Sampling

The samples were respectively collected on days 7, 14 and 28. For each sampling time point, 8 broilers per group were randomly selected and then killed by jugular exsanguination. The small intestines were extracted, and segments (1 cm in length) of the mid-duodenum, jejunum and ileum were excised and lightly rinsed with sterile PBS to remove the intestinal digesta, which were then fixed in 4% (v/v) paraformaldehyde for morphological examination (performed by Wuhan Goodbio technology Co., Ltd., Wuhan, China). The ileum mucosae were scraped off with a glass slide, rapidly frozen in liquid nitrogen tank, and then transferred to − 80 °C freezer for storage. The bilateral ceca were split with sterile scissors and forceps. Then, the caecal digesta were scraped to frozen tubes and stored at − 80 °C for metabolomics analysis.

### Intestine morphological analyses and observation

The small intestine slides were photographed by a light microscope (Nikon Corp., Tokyo, Japan). Intestinal morphological parameters of each slide were calculated based on the average of five villus crypt units with intact lamina propria [20]. Villus height was measured from the villus tip to the villus-crypt junction, and the crypt depth was defined as the length from the villus-crypt junction to the base of the crypt. Furthermore, the villus height-to-crypt depth ratio (V/C) was obtained according to the means of villus height and crypt depth.

### Quantitative real-time PCR analysis

Total RNA in the ileal mucosa was isolated using the MiniBEST Universal RNA Extraction Kit (Takara Bio, Dalian, Liaoning, China). RNA quantity and quality were determined using a spectrophotometer (NanoDrop-2000, Thermo Fisher Scientific, MA, USA). cDNA was synthesized using SuperScript™ III Reverse Transcription in the presence of random primers and an RNase inhibitor (Invitrogen, Carlsbad, USA) according to the manufacturer’s instructions. Gene-specific primers for zonula occludens-1 (*ZO1*), zonula occludens-2 (ZO2), claudin-1 (*CLDN1*) and claudin-2 (*CLDN2*) were designed with Primer Premier 6.0 (Table 1) and commercially produced in Tsingke (Beijing, China). The RT-PCR assays were performed in triplicate on an ABI Prism 7500 Real-Time PCR Detection System (Applied Biosystems, Foster City, CA, USA). The reaction protocol was as follows: 1 min at 95 °C, followed by 40 cycles of 15 s denaturation at 95 °C, and 25 s annealing at 63 °C. This was followed by a melting curve analysis to confirm the specificity and reliability of the PCR products. The relative mRNA expression was obtained using the 2^−∆∆Ct^ method, as previously described [21], after normalization with the expression of glyceraldehyde 3-phosphate dehydrogenase (GAPDH).

**Table 1:** Primers for gene expression analysis using real-time PCR

Gene name	Primer sequences (5′→3′)	GenBank accession	Expected size, bp
*ZO1*	F: CCACTGCCTACACCACCATCTC R: CGTGTCACTGGGGTCCTTCAT	XM_015278975.1	138
*ZO2*	F: CCCAGTGGTTTCCCATTGTAGTC R: GAACACAGCCTTTGTCTCATCGT	XM_015280242.1	79
*CLDN1*	F: GCATGGAGGATGACCAGGTGA R: GAGCCACTCTGTTGCCATACCAT	NM_001013611.2	117
*CLDN2*	F: CCTACATTGGTTCAAGCATCGTGA R: GATGTCGGGAGGCAGGTTGA	NM_001277622.1	131
*GAPDH*	F: CAGAACATCATCCCAGCGTCCA R: ACGGCAGGTCAGGTCAACAA	NM_204305.1	135

### Sample preparation for GC-TOF/MS analysis

Approximately 50 ± 1 mg caecal content was extracted with 400 μL miscible liquid (V _methanol_: V _chloroform_ = 3:1) and placed into 2 mL epoxy epoxide (EP) tubes. Subsequently, 10 μL of L-2-Chlorophenylalanine (0.5 mg/mL stock in dH_2_O, internal standard) was added and mixed by vortexing for 30 s. The mixed sample was homogenized in a ball mill for 4 min at 40 Hz, which was followed by ultrasonic treatment for 5 min (incubated in ice water). After centrifugation at 12000 r/min for 15 min, 350 μL of supernatant was injected into a fresh 1.5 mL EP tubes. The liquid was completely dried in a vacuum concentrator without heating, and then 50 μL methoxy amination hydrochloride (20 mg/mL in pyridine) was added and mixed lightly. The mixed sample was incubated for 30 min at 80 °C. Afterwards, 70 μL of the BSTFA regent (1% TMCS, v/v) was added to each sample, followed by incubation for 1.5 h at 70 °C. Finally, the processed samples would be subjected to GC-TOF/MS analysis.

### GC-TOF/MS analysis

Pretreated caecal content samples were analyzed using an Agilent 7890 gas chromatograph system (Agilent, USA) coupled with a Pegasus HT time-of-flight mass spectrometer (LECO, USA). In addition, a DB-5MS capillary column (30 m × 250 μm × 0.25 μm; J&W Scientific, Folsom, CA, USA) was used in the determination to separate the derivatives. In splitless mode, the injection volume was set at 1 μL, and the solvent delay time was 4 min. Helium served as the carrier gas running in the column at a constant flow rate of 1 mL/min. The initial temperature was programmed at 50 °C for 1 min, increased at 10 °C/min to 290 °C, and subsequently maintained for 8 min at 290 °C. The injection, transfer line, and ion source temperatures were 280, 280, and 250 °C, respectively. The analysis was carried out in electron impact mode at − 70 eV. The mass spectral data were obtained in full scan mode from m/z 50 to 500 at a rate of 20 spectra per second after a solvent delay of 366 s [22].

### Data analysis

The GC-TOF/MS raw data were first analyzed by Chroma TOF 4.3X software and LECO-Fiehn Rtx5 database, including raw peaks extraction, data baseline filtering and calibration, peaks alignment, deconvolution analysis, peaks identification, and integration of the peak area [23]. Subsequently, the resulting normalized data were input into the SIMCA 14.1 software package (Umetrics AB, Umea, Sweden). After mean centralizing and unit variance scaling, multivariate analyses, such as principal component analysis (PCA) and orthogonal partial least squares discriminant analysis (OPLS-DA), were performed to visualize metabolic differences between the two groups. Finally, the differentially expressed metabolites were characterized and recognized by searching the online Kyoto Encyclopedia of Genes and Genomes (KEGG, http://www.genome.jp/kegg/). In addition, a free and web-based tool, MetaboAnalyst (http://www.metaboanalyst.ca/), which uses the high-quality KEGG metabolic pathway as the backend knowledgebase, was used for pathway analysis.

The results of intestinal morphology and the relative mRNA expression of tight junction proteins were expressed as means ± SEM. The *P* values < 0.05 were considered as the significance threshold, and *P* values < 0.01 were defined as an extremely significant differences based on an independent-sample *t*-test. All analyses were conducted using SPSS (Version 22.0).

## Results

### Intestine morphology

**Fig. 1:**
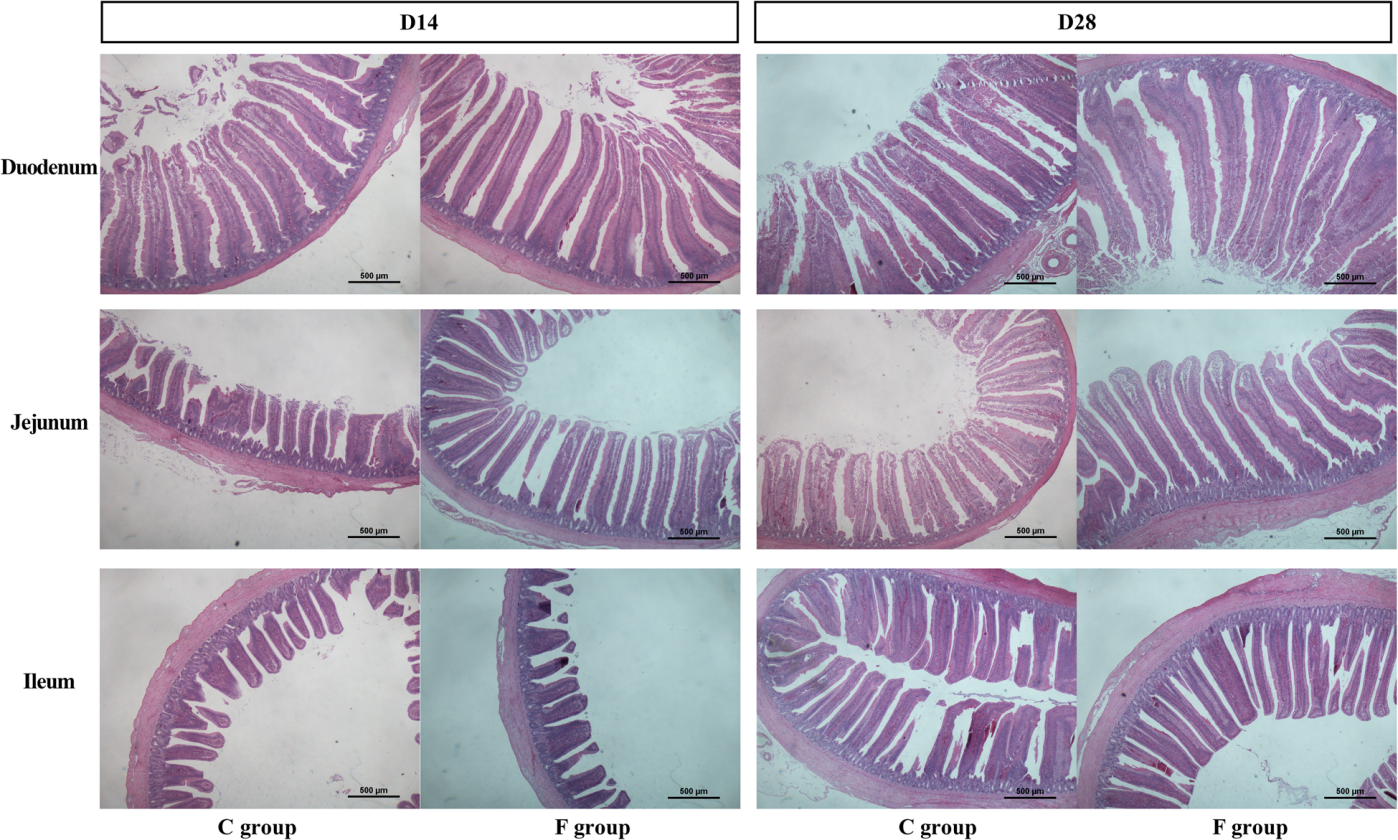
Histomorphology of small intestine of 14-day-old and 28-day-old broilers. C group: control group; F group: fermentation broth group

**Fig. 2:**
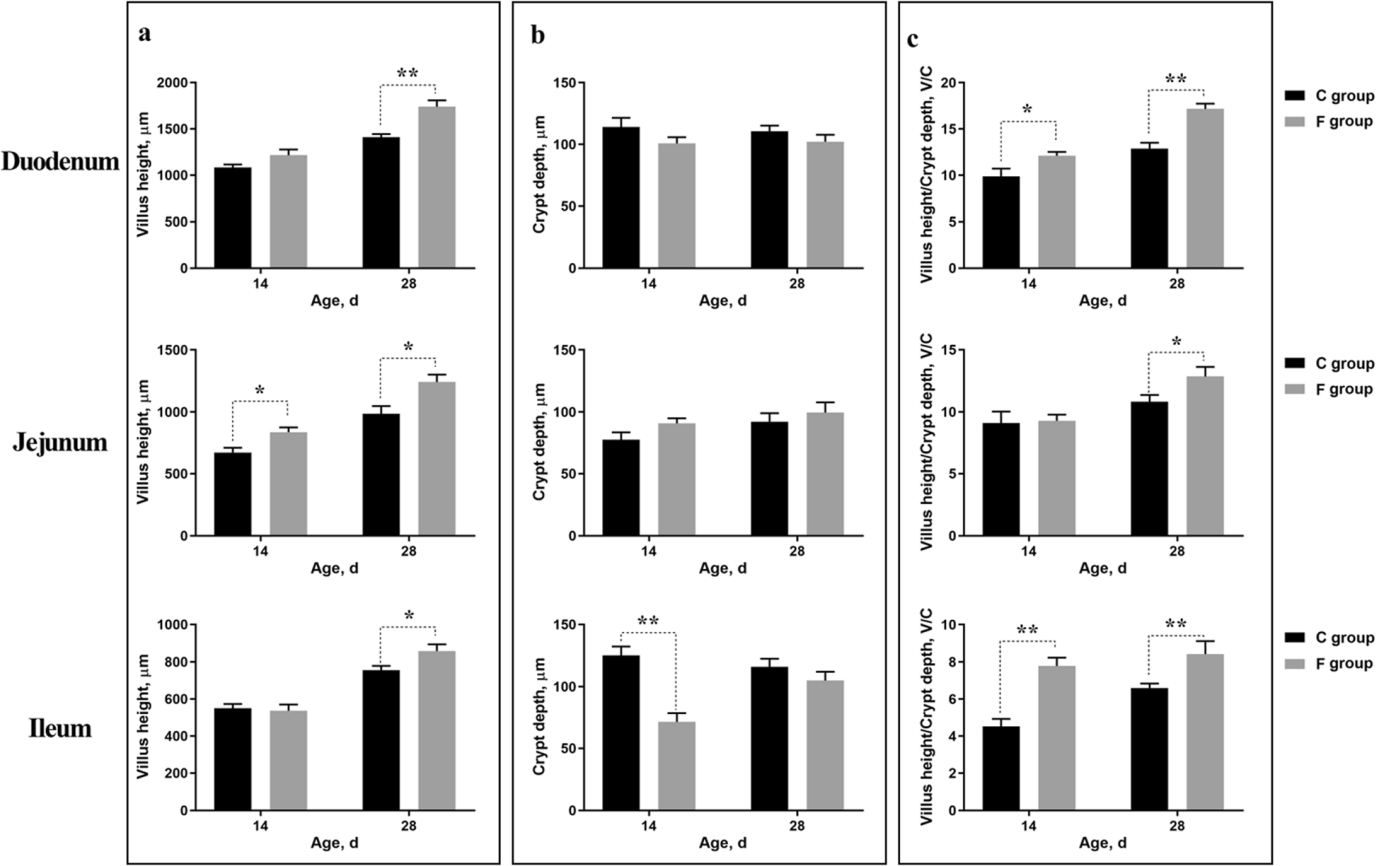
Effect of early inoculation on villus height and crypt depth of the small intestines of 14-day-old and 28-day-old broilers. **a** Villus height of the duodenum, jejunum and ileum. **b** Crypt depth of the duodenum, jejunum and ileum. **c** Ratio of villus height to crypt depth (V/C) of the duodenum, jejunum and ileum. The data are presented as means ± SEM (*n = 8*). Asterisks (* and **) represent significant differences with *P* < 0.05 and *P* < 0.01, respectively. C group: control group; F group: fermentation broth group

The light micrograph of small intestine morphology is exhibited in Fig. 1. Early inoculation increased the villus heights of the duodenum, jejunum and ileum. Moreover, this result was statistically confirmed by the data shown in Fig. 2. The villus height of jejunum was markedly increased in the F group on days 14 (*P* < 0.05) and 28 (*P* < 0.05). In addition, early inoculation significantly increased the villus heights of duodenum (*P* < 0.01) and ileum (*P* < 0.05) on day 28. However, early inoculation had no significant effect on crypt depth, except for in the ileum on day 14. It is worth noting that the ratio of villus height to crypt depth (V/C) in the small intestine in the F group was significantly increased when compared with the C group (*P* < 0.05).

### Gene expression of tight junction proteins

**Fig. 3:**
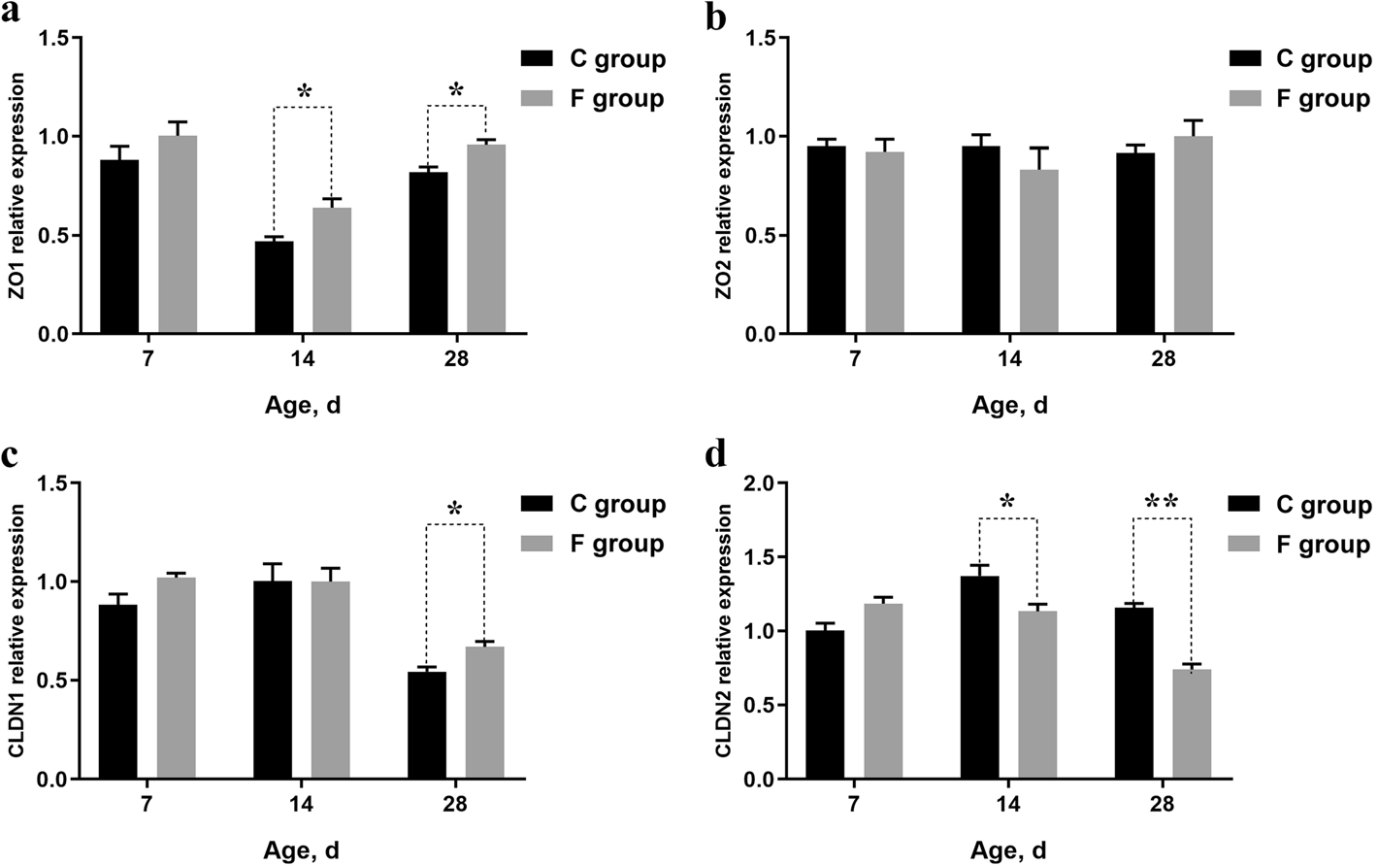
Relative gene expression of *ZO1* (**a**), *ZO2* (**b**), *CLDN1* (**c**) and *CLDN2* (**d**) in the ileum of 7-day-old, 14-day-old and 28-day-old broilers. The data are presented as means ± SEM (*n = 5*). Asterisks (* and **) represent significant differences with *P* < 0.05 and *P* < 0.01, respectively. *ZO1*: zonula occludens-1; *ZO2*: zonula occludens-2; *CLDN1*: claudin-1; *CLDN2*: claudin-2. C group: control group; F group: fermentation broth group

To further investigate the effect of early inoculation on the intestinal barrier integrity, the relative gene expression levels of tight junction proteins *CLDN1*, *CLDN2*, *ZO1*, and *ZO2* in ileum were measured (Fig. 3). Early inoculation significantly upregulated the ileum *ZO1* mRNA expression on days 14 and 28 (*P* < 0.05), and the ileum *CLDN1* mRNA expression on day 28 (*P* < 0.05). In contrast, the relative gene expression levels of *CLDN2* on days 14 (*P* < 0.05) and 28 (*P* < 0.01) were dramatically decreased in the F group. However, no significant differences were found regarding the relative expression of ileum *ZO2* between the two groups at either 14 or 28 d.

### Metabolites profiling using GC-TOF/MS

The GC-TOF/MS platform was used to study the response of intestinal metabolomics profiles to early inoculation with caecal fermentation broth. Total ion chromatograms (TIC) of caecal content samples from the C group and the F group on days 7 and 14 are displayed in Additional file 1: Figure S1. A total of 457 valid peaks were extracted after preprocessing of the raw data; among the peaks, 179 compounds were relatively quantified, 102 were marked “unknown” and 176 were labeled “analyte” when referring to the LECO-Fiehn Rtx5 Metabolomics Library.

### Statistical comparison of metabolites

**Fig. 4:**
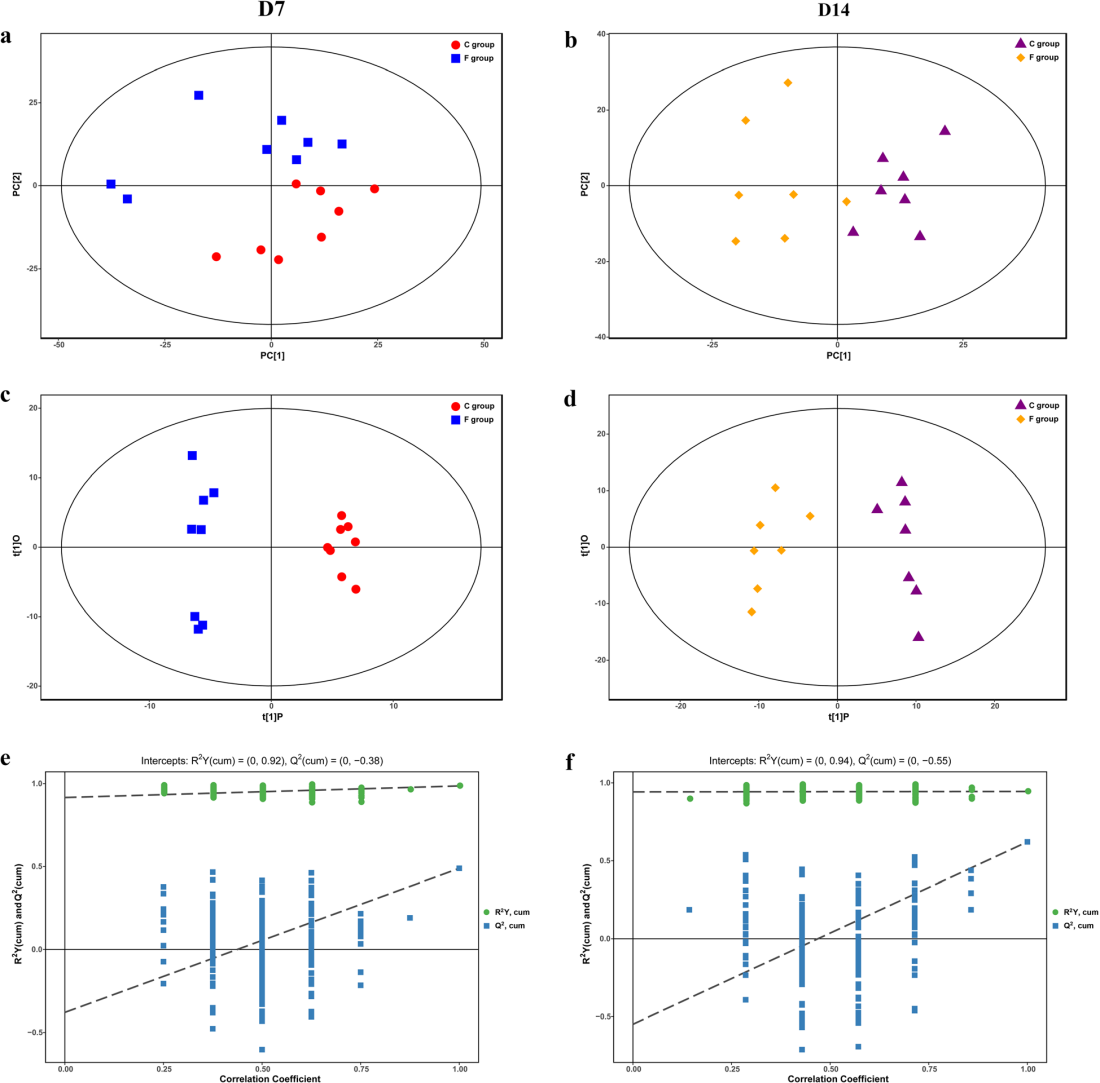
PCA score plots (**a** and **b**), OPLS-DA score plots (**c** and **d**) and OPLS-DA corresponding validation plots (**e** and **f**) resulting from the GC-TOF/MS metabolite profiles of the caecal contents of 7-day-old and 14-day-old broilers. PCA: principal component analysis; OPLS-DA: orthogonal partial least squares discriminant analysis. C group: control group; F group: fermentation broth group

The differences in the metabolomics profiles between the C group and the F group by the multivariate analysis are displayed in Fig. 4. The overall changes in metabolic physiology were readily observed by the unsupervised PCA of the entire set of analytes tested. The PCA score plots of the C group and the F group showed a general separation trend not only on day 7 but also on day 14 (Fig. 4a and b). None of the samples went beyond the 95% Hotelling’s T-squared ellipse, suggesting that there was no outlier among the analyzed samples. To obtain a better understanding of early intervention responsible for classification and a higher level of group separation, an OPLS-DA was employed to elucidate the different metabolic patterns. The OPLS-DA plots (Fig. 4c and d) showed markedly separated clusters between the C group and the F group on days 7 and 14. In addition, the OPLS-DA model was tested by a random-permutations analysis to confirm its validity and robustness (Fig. 4e and f). The 200 permutation texts result showed that the respective R^2^Y and Q^2^ intercept values were 0.92 and − 0.38 on day 7, and 0.94 and − 0.55 on day 14. In general, when the corresponding Q^2^ value is less than 0, the statistical model is determined to be valid with a low risk of over fitting [24]. Therefore, the lower values of the Q2 intercept in our results suggested that the robustness of the models was suitable for metabolomics analysis.

### Identification of significantly different metabolites

**Fig. 5:**
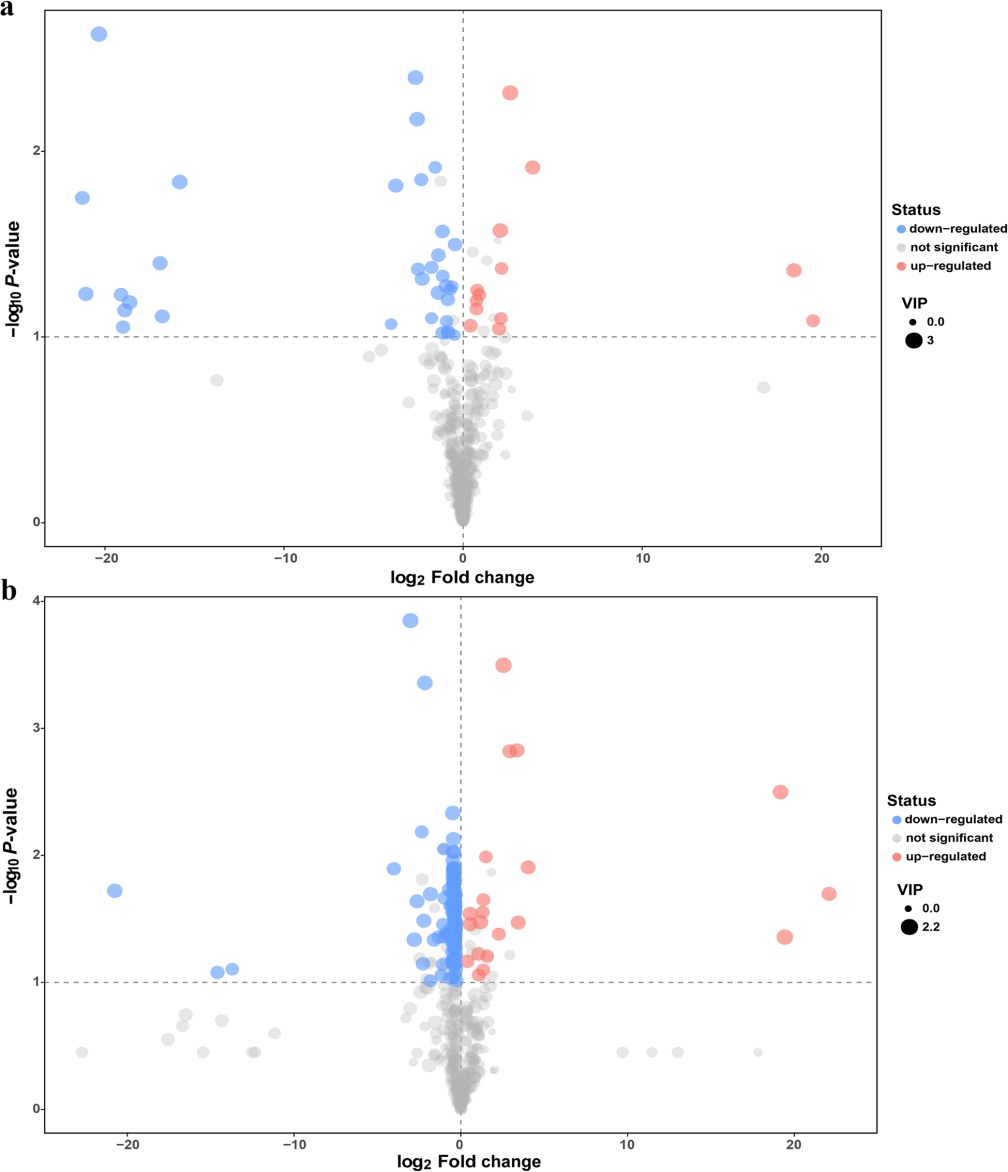
Volcano plots of caecal metabolites of 7-day-old (**a**) and 14-day-old (**b**) broilers. The red dot represents a significantly different metabolite that is more concentrated in the F group, while the blue dot represents a metabolite that was mainly enriched in the C group. The dot size indicates the variable importance in the projection (VIP) value. Fold Change: mean value of peak area obtained from the F group/mean value of peak area obtained from the C group

**Fig. 6:**
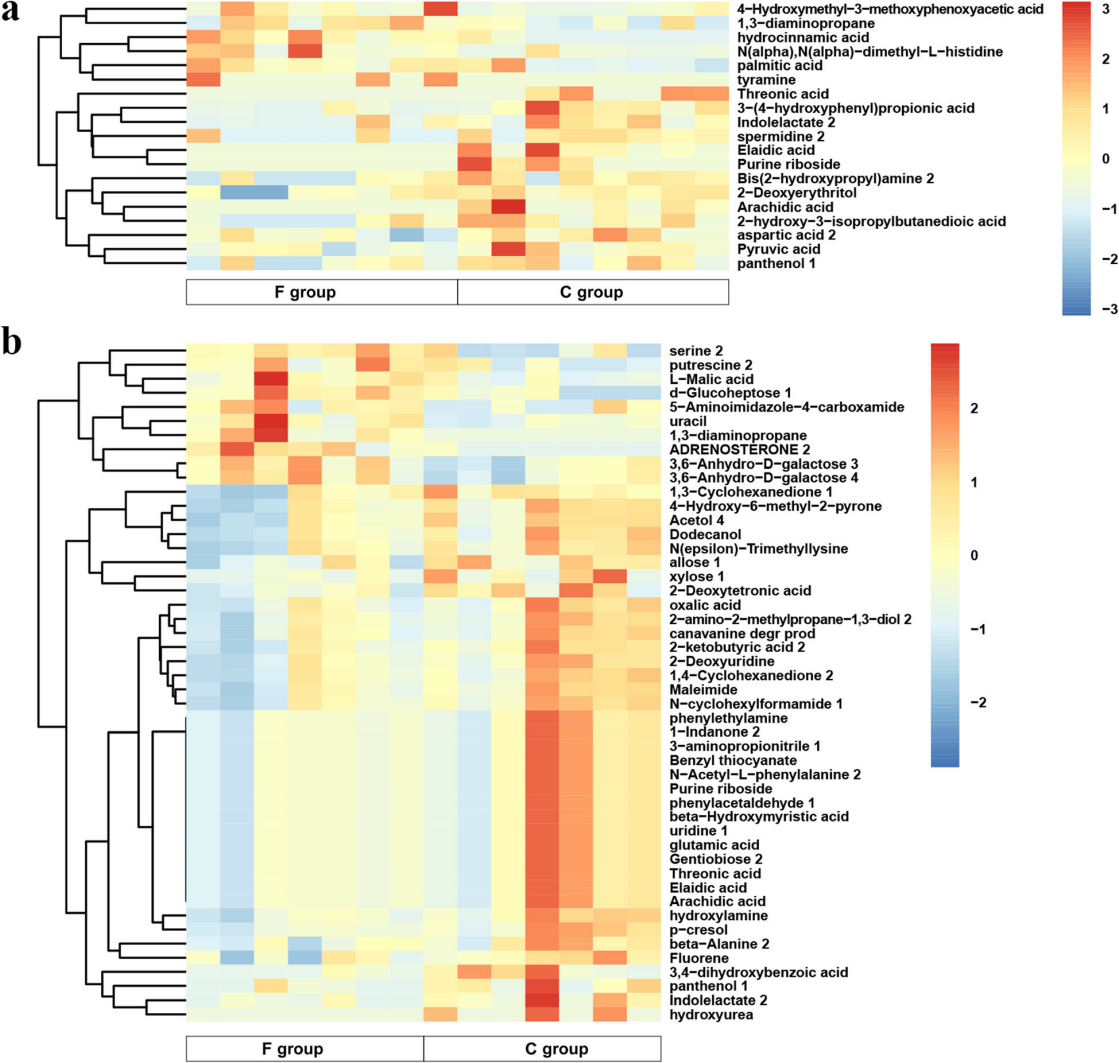
Hierarchical clustering analysis for the significantly different metabolites based on average clustering algorithm with Pearson distance. **a** 7-day-old broilers. **b** 14-day-old broilers. C group: control group; F group: fermentation broth group

The differentially expressed metabolites were recognized according to the following assessment criterion: the variable importance for the projection (VIP) values > 1.0 in OPLS-DA model and *P* values < 0.05 in Student’s t-test. Relative to the C group, the relative levels of 47 and 166 metabolites were significantly altered in the F group on days 7 and 14, respectively. As shown in Fig. 5a, 13 metabolites in the F group were upregulated, and 34 metabolites were downregulated on day 7. Similar results were found on day 14, with 20 upregulated metabolites and 146 downregulated metabolites (Fig. 5b). Hierarchical clustering together with a heatmap was performed to reveal the distinct characteristics of the differentially expressed metabolites (excluding the 28 from 47 total differentially expressed metabolites that were unidentified on day 7, and the 118 from 166 on day 14) based on the relative levels of the identified metabolite. After annotation, it was observed that 6 compounds on day 7 and 10 compounds on day 14 exhibited higher concentrations in the F group than that in the C group. By contrast, 13 and 38 compounds were respectively substantially downregulated on days 7 and 14 (Fig. 6 and Additional file 2: Table S1). More importantly, we found that the level of 1,3-diaminopropane in the F group was significantly increased not only on day 7, but also on day 14. It was the only significantly different metabolite that was upregulated at both test ages.

### Characterization of significantly different metabolic pathways that responded to early inoculation

**Fig. 7:**
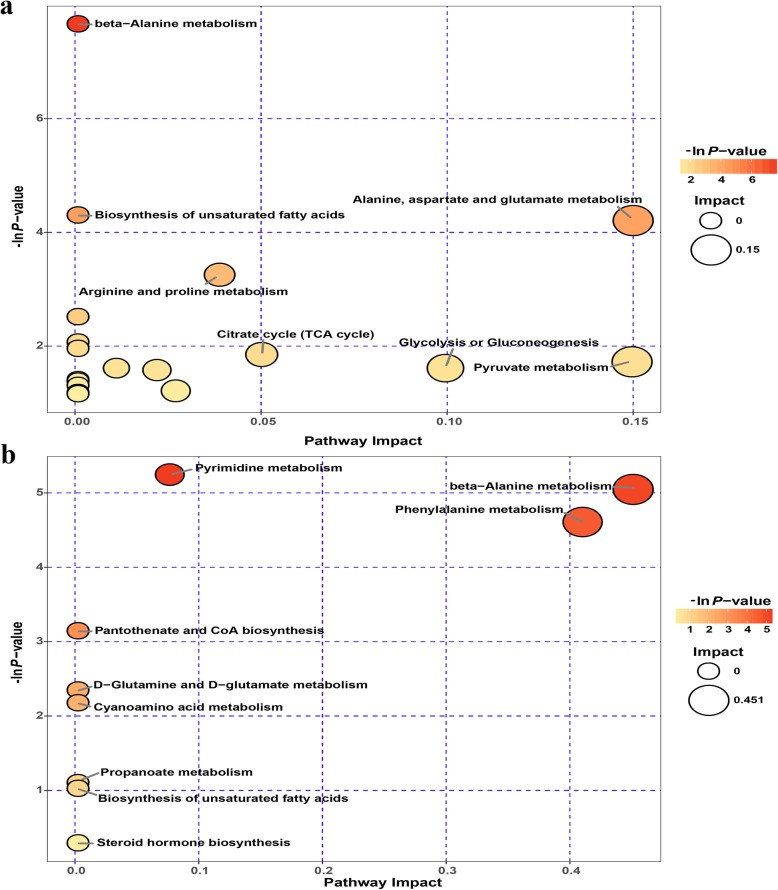
Metabolome view map of the significantly different metabolites. Significantly changed pathways based on enrichment and topology analysis are shown. The *X*-axis represents the pathway impact, and the *Y*-axis represents the pathway enrichment. Larger sizes and darker colors represent higher pathway impact values and higher pathway enrichment, respectively. **a** 7-day-old broilers. **b** 14-day-old broilers

To explore the significantly different metabolic pathways that responded to early inoculation, we imported the significantly different metabolites (Additional file 3: Table S2) into KEGG. Consequently, 18 and 9 metabolic pathways were respectively identified between the C group and the F group on days 7 and 14 (Additional file 4: Table S3). As shown in the metabolome view map (Fig. 7), the relevant metabolic pathways were illustrated using both –ln *P*-value and pathway impact scores as criteria. These pathways mainly include amino acid metabolism (beta-alanine, alanine, aspartate, glutamate, arginine and proline metabolism), energy metabolism (biosynthesis of unsaturated fatty acids, citrate cycle and pyruvate metabolism) and glycolysis or gluconeogenesis on day 7 (Fig. 7a), and amino acids metabolism (beta-alanine and phenylalanine metabolism), pantothenate and CoA biosynthesis, and pyrimidine metabolism on day 14 (Fig. 7b).

## Discussion

The objective of this study was to determine whether inoculation of fermentation broth at 2 h post-hatch affects the small intestine morphology, expression levels of tight junction-proteins in the ileum, and the caecal metabolomics profiling of broilers. Maintaining optimal health of animals is highly dependent on the complete intestine morphology and nutrient metabolism in the complex intestinal system. We know that an optimized environment in the gut is critical to host health. It has been shown in some published papers that the changes of intestinal environment in early life can alter the colonization and development of gut microbiota, and then regulate the subsequent development and health of animals [25, 26]. In this regard, inoculation with caecal fermentation broth for broilers at the early stage of life could be used as a new regulation strategy for poultry health relative to the traditional nutrition regulation.

In the present study, we used the broiler model to investigate the response of the intestinal physical barrier to early inoculation. The results showed that early inoculation with caecal fermentation broth can improve small intestinal morphology, as indicated by increased villi length as well as villus height to crypt depth ratios in the duodenum, jejunum and ileum. The intact intestinal structure is important for animal digestion and absorption, which is closely related to the morphological characteristic of small intestinal villus length and crypt depth [27, 28]. A previous study revealed that the villus height is correlated with the absorptive capability of the small intestine, elevations in villus height would contribute to nutrient transport [29]. Similarly, the morphology parameters measured in our study increased significantly, particularly villus height, suggesting that early inoculation could improve the absorption function of small intestine. Consistently, this can be used to explain our previous study, which found that early inoculation with caecal fermentation broth had a positive effect on the growth performance of broilers [11].

In poultry studies, the lack of appropriate commercial avian antibodies makes it impossible to assess the effects at the protein level. Hence, a qRT-PCR technique was performed in our experiment to illuminate the effect of early inoculation on the relative gene expression of tight junction proteins, namely, CLDN1, CLDN2, ZO1 and ZO2 [30]. We observed that early inoculation promoted upregulation of the expression of tight junction protein CLDN1 on day 28, and ZO1 on days 14 and 28. However, the relative expression level of tight junction protein CLDN2 was dramatically decreased in the intervened broilers on days 14 and 28. As one of the most important components of the intestinal epithelial barrier, CLDN1 can effectively prevent harmful substances from reaching the surface of the epithelial cells [31]. A previous study showed that the high expression of CLDN1 could result in increased epithelial tightness and decreased solute permeability [32]. In contrast, CLDN2 is a pore-forming tight junction protein [33]. The upregulation of CLDN2 expression has been coincidentally reported just in inflammatory bowel disease, and it is usually considered an indicator of inflammation [34]. The zona occludens family, including ZO1 and ZO2, is a set of scaffolding proteins that is an essential part of the tight junctions in cytoplasmic plaques [30]. Taken together, the increased gene expression of CLDN1, and ZO1 and the decreased gene expression of CLDN2 in the intervened broilers point to an enhancement of the intestinal barrier by early inoculation.

The knowledge of the interaction between microbiota metabolism and host health is truly beneficial for effective regulation of the growth and health of birds. The phenotype of an organism and its reaction to environmental perturbations are usually closely related to its metabolism, and the development of metabolome technology in recent years provides a unique method to examine the physiological status of the biological system [26]. To further explore the effect of early inoculation on intestinal physiological function, a GC-TOF/MS-based metabolomics method was applied in the current study to quantitatively detect small molecular metabolites in broilers caecum and identify relevant pathways. The intestinal metabolomics profiles of the F group remarkably differed from those of the C group. In total, 47 metabolites were altered followed early inoculation on day 7, and 166 were altered on day 14. More differentially expressed metabolites detected on day 14 were attributed to the increased metabolic capacity of broilers with age. However, it should be noted that not all of these metabolites at the age of 7 and 14 days presented the same increasing or decreasing trend. Among all the 47 and 166 differentially expressed metabolites identified by multivariate statistical analysis on days 7 and 14, respectively, only one compound, namely 1,3-diaminopropane, was continuously elevated following early inoculation on days 7 and 14. Using an untargeted metabolomics analysis, a previous study also found that 1,3-diaminopropane was significantly increased in the recipient piglets following fecal microbiota transplantation [35], which is in line with our result. Therefore, 1,3-diaminopropane was selected as a biomarker, suggesting that this compound could be used as a biomarker of environmental exposure of animal gut at an early stage of life. Previous scientific evidence indicated that 1,3-diaminopropane, a biologically significant diamine, is metabolized by amino acid [36]. As a type of polyamines, 1,3-diaminopropane is considered to play various biological roles in gene expression, cell death and cell stress protection [37, 38]. This notion is supported by the enhanced intestinal morphology and the increased expression levels of tight junction proteins CLDN1 and ZO1 in our study.

Subsequently, we conducted metabolic pathway analyses to put all of the differentially expressed metabolites into the context of connected metabolic pathway networks. Several key metabolic pathways were identified to respond to early inoculation with caecal fermentation broth. After analysis of these metabolic pathways, beta-alanine metabolism and biosynthesis of unsaturated fatty acids were considered as significantly impacted metabolic pathways, which were not only in the metabolome view map on day 7 but also on day 14. During beta-alanine metabolism, the level of 1,3-diaminopropane in the F group was higher than that of the C group, resulting in a decreased beta-alanine level in the intervened broilers. An increasing number of reports have now demonstrated that an accumulation of beta-alanine was detected in breast cancer [39, 40], suggesting that elevated levels of beta-alanine might be a disadvantage to human health. Therefore, this alternation in beta-alanine metabolism in the present study may have a positive effect on the growth and health of the intervened broilers. However, the role of beta-alanine in health remains to be further studied. Previous reports have revealed that unsaturated fatty acids plays a vital role in inflammation and regulation of immunity [41, 42]. Based on our results, biosynthesis of unsaturated fatty acids was found to be accelerated by early inoculation with caecal fermentation broth. The elevated biosynthesis of unsaturated fatty acids in broilers probably contributed to trigger unsaturated fatty-acid-dependent killing of pathogenic bacteria, which is in line with the significantly decreased abundance of pathogenic bacteria in our previous 16S rRNA gene sequencing results [11]. In the present study, characterization of metabolism induced by early intestinal environmental exposure could reveal the major regulated metabolic pathways for broilers’ growth and health, but the specific metabolic mechanism still needs further investigation.

## Conclusion

Taken together, our study demonstrated that early inoculation with caecal fermentation broth not only altered intestinal morphological parameters but also changed the caecum metabolic profile characterization of broilers. The metabolomics results showed that beta-alanine metabolism and biosynthesis of unsaturated fatty acids were the key metabolic pathways affected by early inoculation in which the significantly different metabolites were mainly enriched. The compound 1,3-diaminopropane was selected as a potential biomarker that responded to administration with caecal fermentation broth in the early stage of broilers. Additionally, the changes in the intestinal environment induced by early inoculation improved small intestine morphology and the gene expression of tight junction proteins in the ileum. These findings provide insight into intestinal metabolomics and provide an environmental intervention strategy for broiler growth and health.

## Supplementary information

**Additional file 1: Figure S1.** Typical GC-TOF/MS TIC chromatograms of caecal content samples from the C group and the F group. The green and blue lines represent the C group on days 7 and 14, the red and black lines represent the F group on days 7 and 14.

**Additional file 2: Table S1.** The hierarchical clustering data matrix for the C group and the F group on days 7 and 14.

**Additional file 3: Table S2.** Significantly different metabolites between the C group and the F group. The red text indicates the significantly different metabolite that is more abundant in the F group, while the blue text represents the higher concentration of significantly different metabolite in the C group.

**Additional file 4: Table S3.** Pathway analysis for the C group and the F group on days 7 and 14.

## Data Availability

All data generated or analyzed during this study are available from the corresponding author on reasonable request.

## Abbreviations

CLDN1: Claudin-1
CLDN2: Claudin-2
CoA: Coenzyme A
EP: Epoxy epoxide
GC-TOF/MS: Gas chromatography time-of-flight/mass spectrometry
GIT: Gastrointestinal tract
OPLS-DA: Orthogonal partial least squares discriminant analysis
PBS: Phosphate buffered saline
PCA: Principal component analysis
qRT-PCR: Quantitative real-time polymerase chain reaction
TIC: Total ion chromatogram
V/C: The villus height-to-crypt depth ratio
VIP: Variable-importance-projection
ZO1: Zonula occludens-1
ZO2: Zonula occludens-2
