# Supplementary figures and images for: Early inoculation with caecal fermentation broth alters small intestine morphology, gene expression of tight junction proteins in the ileum, and the caecal metabolomic profiling of broilers
Source: J Anim Sci Biotechnol. 2020 Jan 15;11:8. doi: 10.1186/s40104-019-0410-1 (PMC6961334; doi:10.1186/s40104-019-0410-1)

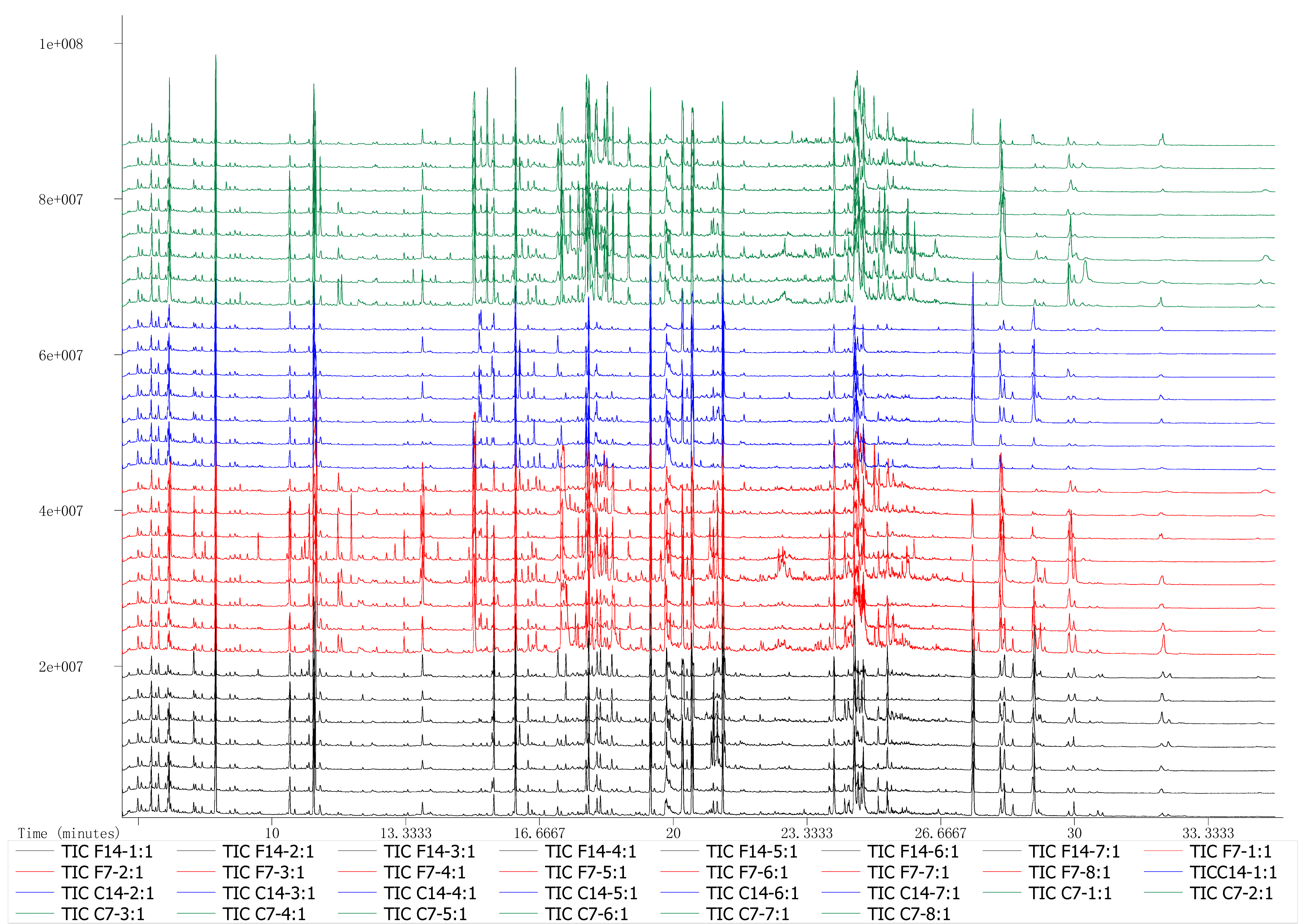

Supplement: Supplementary file 1 — Additional file 1: Figure S1. Typical GC-TOF/MS TIC chromatograms of caecal content samples from the C group and the F group. The green and blue lines represent the C group on days 7 and 14, the red and black lines represent the F group on days 7 and 14. [file 40104_2019_410_MOESM1_ESM.tif]
